# Perceived Rabbit Care Knowledge Does Not Consistently Align with Owner-Reported Husbandry Practices: A Survey of Pet Rabbit Owners in Croatia

**DOI:** 10.3390/ani16121830

**Published:** 2026-06-14

**Authors:** Magdalena Neva Oelsner, Ivana Sabolek, Jurica Novak, Gordana Gregurić Gračner

**Affiliations:** 1 Faculty of Veterinary Medicine, University of Zagreb, Heinzelova 55, 10000 Zagreb, Croatia; moelsner@vef.hr (M.N.O.); ggracner@vef.unizg.hr (G.G.G.); 2 Center for Informatics and Computing, Ruđer Bošković Institute, Bijenička Cesta 54, 10000 Zagreb, Croatia

**Keywords:** pet rabbit, animal welfare, husbandry practices, owner knowledge, perceived knowledge, knowledge–practice gap, companion animals, survey study, Croatia

## Abstract

The welfare of pet rabbits is frequently compromised despite increasing recognition of their complex behavioural and physiological needs. Although owner knowledge is considered an important determinant of animal care, the relationship between perceived knowledge and actual husbandry practices remains poorly understood. This study aimed to (i) describe husbandry practices and owner-reported knowledge among pet rabbit owners in Croatia and (ii) assess whether higher perceived knowledge is associated with greater alignment with recommended husbandry practices. Data were collected using an online questionnaire distributed to rabbit owners (*n* = 529). The study assessed owner-reported husbandry practices and perceived knowledge but did not include direct assessments of rabbit health or welfare. Although most respondents reported high levels of knowledge regarding rabbit care (85.6%), this was not consistently reflected in implemented husbandry practices. Higher perceived knowledge was significantly associated (*p* < 0.05) with environmental enrichment, housing type, and cleaning frequency, but these associations were generally weak to moderate. In contrast, perceived knowledge showed little or no association with several key welfare-related practices, including social housing and access to free movement, while only limited associations were observed for feeding practices. These findings reveal a substantial knowledge–practice gap, suggesting that confidence in rabbit care knowledge does not necessarily translate into appropriate husbandry. Therefore, educational approaches focused solely on information provision may be insufficient to ensure the implementation of recommended husbandry practices.

## Simple Summary

Pet rabbits are popular companion animals, but previous studies have reported that many rabbits are kept under husbandry conditions that may not fully meet their behavioural and physiological needs. A survey of 529 rabbit owners in Croatia showed that, although most owners considered themselves knowledgeable about rabbit care, this was not consistently reflected in their everyday husbandry practices. Many rabbits were housed individually despite recommendations favouring compatible social housing, and even owners reporting high levels of knowledge did not consistently report husbandry practices aligned with current welfare recommendations. Higher perceived knowledge was associated with some positive husbandry practices, such as environmental enrichment and housing type, but these associations were generally weak. The findings suggest that confidence in rabbit care knowledge does not necessarily translate into appropriate care and highlight the need for educational approaches that better support the practical implementation of recommended husbandry practices.

## 1. Introduction

Rabbits (*Oryctolagus cuniculus*) have become increasingly popular companion animals across Europe, including Croatia [1,2,3,4,5]. Despite this growing popularity, substantial welfare concerns in pet rabbits continue to be reported [6,7]. Rabbits are highly sensitive animals with complex behavioural, environmental, nutritional, and social needs, and inadequate husbandry may compromise both their physical and psychological welfare, contributing to behavioural, dental, gastrointestinal, and musculoskeletal disorders [8,9,10,11]. Furthermore, as prey animals, rabbits often mask signs of pain and illness, making welfare problems difficult for owners to recognise at an early stage and potentially delaying appropriate intervention [12].

Rabbits are frequently perceived as low-maintenance pets and suitable companion animals for children, despite their complex environmental, behavioural, and social requirements [13,14,15,16]. Inadequate understanding at the time of acquisition may therefore contribute to inappropriate husbandry practices and compromised welfare [17]. These challenges may be further exacerbated by lower familiarity with rabbit-specific behavioural and husbandry requirements among both owners and veterinary professionals compared with more traditional companion animals, such as dogs and cats [18,19,20]. Importantly, welfare outcomes are likely influenced not only by owner knowledge but also by owner attitudes and beliefs, suggesting that the interpretation and practical application of information play a key role in everyday husbandry practices [21].

However, the relationship between owner knowledge and rabbit welfare may be more complex than a simple lack of information. A distinction should be made between perceived and actual knowledge [22], as owners may express confidence in their understanding of rabbit care while their husbandry practices do not consistently align with evidence-based recommendations. Such discrepancies may represent an important but underexplored welfare risk in pet rabbits. Importantly, the present study focuses on owner-reported husbandry practices considered relevant to rabbit welfare according to current recommendations, rather than direct assessments of animal welfare outcomes.

Although previous studies have reported deficiencies in rabbit owner knowledge and husbandry practices [6,17,23], no study has specifically examined whether owner-reported knowledge is reflected in actual husbandry practices among pet rabbit owners in Croatia. Understanding this potential knowledge–practice discrepancy may help improve educational and welfare-oriented interventions for pet rabbits.

Therefore, the aims of this study were to (i) describe current husbandry practices and owner-reported knowledge among pet rabbit owners in Croatia and (ii) assess whether higher levels of perceived knowledge are associated with more appropriate husbandry practices. We hypothesised that higher owner-reported knowledge would not necessarily correspond to more appropriate husbandry practices, indicating a potential gap between perceived knowledge and its practical application.

## 2. Materials and Methods

The study was conducted in accordance with ethical principles for research involving human participants. Participation was voluntary and anonymous, and informed consent was obtained from all respondents before questionnaire completion. Participants were provided with information regarding the purpose of the study, the use of the collected data, and their right to withdraw at any time before submission. No personally identifiable information was collected.

### 2.1. Survey Instrument

Data were collected using a structured online questionnaire developed in Google Forms (Google LLC, Mountain View, CA, USA). The questionnaire was designed based on relevant literature on rabbit welfare, husbandry practices, and owner knowledge [3,16,17,23] and adapted to address the specific aims of the present study.

The questionnaire consisted of five sections: (1) owner sociodemographic characteristics; (2) rabbit-related characteristics; (3) housing and environmental conditions; (4) feeding practices; and (5) owner-reported knowledge and sources of information. All questions were closed-ended.

The instrument was designed to capture both perceived (self-reported) knowledge of rabbit care and self-reported husbandry practices, thereby enabling assessment of potential discrepancies between knowledge and its practical application.

Because the study relied exclusively on owner-reported questionnaire data, husbandry practices could not be objectively verified, and no direct behavioural, clinical, or welfare assessments of the rabbits were performed.

Perceived knowledge was assessed using a three-level self-evaluation scale (low, moderate, high), selected to facilitate respondent interpretation, reduce response burden, and ensure sufficiently large group sizes for categorical analyses. The scale was not formally validated and is therefore interpreted as an exploratory measure of perceived knowledge, consistent with examining discrepancies between perceived knowledge and reported practices. Also, no objective assessment of rabbit care knowledge was performed; therefore, the study specifically examined perceived rather than actual knowledge.

Before distribution, the questionnaire was pilot-tested on 20 rabbit owners to assess clarity, relevance, and comprehensibility. Minor modifications were made based on participant feedback before final dissemination.

### 2.2. Data Collection

The questionnaire was distributed to current rabbit owners via a hyperlink shared on social media platforms (Facebook, Instagram, Twitter, and LinkedIn). Participation in the study was entirely voluntary and anonymous. Before participation, all participants were informed about the purpose of the study and the nature of their involvement. No financial compensation, material benefits, or other incentives were provided.

Data collection was conducted between December 2025 and February 2026. The eligibility criteria required respondents to currently own at least one rabbit at the time of participation.

### 2.3. Data Cleaning

Data were exported from Google Forms into a spreadsheet for further processing and statistical analysis. Prior to analysis, data-cleaning procedures were conducted, including the removal of incomplete questionnaires and responses that did not meet the inclusion criteria (i.e., respondents who did not currently own a rabbit). Potential duplicate submissions were identified based on response similarity and completeness, with only the most complete response retained. A total of 536 responses were received. Following data cleaning, 7 incomplete questionnaires were excluded, resulting in a final dataset comprising 529 eligible respondents.

### 2.4. Statistical Analysis

All statistical analyses were performed using Python 3.12. Data processing was conducted using pandas and NumPy (2.1.3), statistical analyses using SciPy (1.16.1), and data visualisation using Matplotlib (3.10.5). Descriptive statistics are presented as frequencies and percentages.

Associations between categorical variables were assessed using Pearson’s chi-squared test of independence. Fisher’s exact test was applied when expected cell counts were below the assumptions required for the chi-squared test. Statistical significance was set at *p* < 0.05. Associations were assessed using chi-squared tests. Effect sizes were estimated using bias-corrected Cramér’s V and interpreted as negligible (<0.10), weak (0.10–0.20), moderate (0.20–0.40), or strong (>0.40).

## 3. Results

After data cleaning and application of the inclusion criteria, a total of 529 complete and eligible questionnaires were included in the final analysis.

### 3.1. Respondent Demographic Characteristics

**Figure 1:**
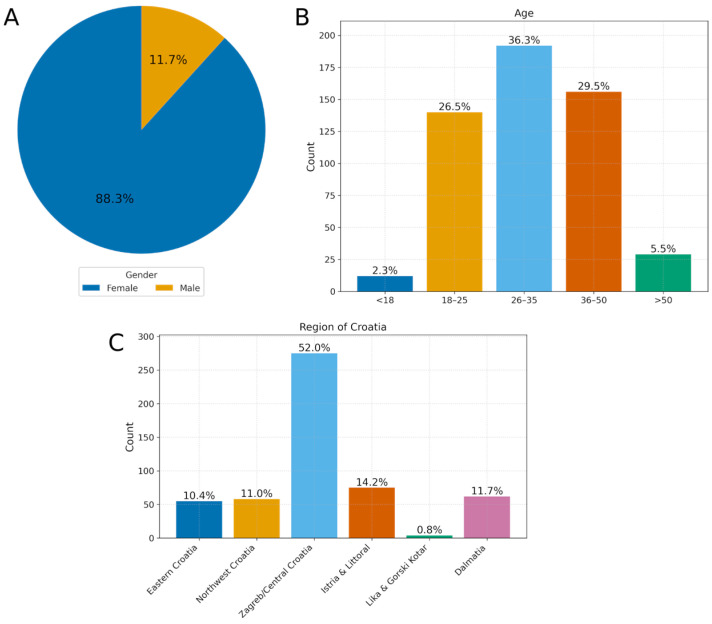
Demographic characteristics of owners, including (**A**) gender, (**B**) age distribution, and (**C**) region of Croatia. Values are presented as percentages.

The respondent sample was predominantly female (88.3%) (Figure 1A). The most represented age group was 26–35 years (36.3%), followed by 36–50 years (29.5%) and 18–25 years (26.5%), while participants younger than 18 years (2.3%) and older than 50 years (5.5%) were least represented (Figure 1B).

Respondents were distributed across all regions of Croatia, with the highest proportion from Zagreb and Central Croatia (52.0%). The lowest representation was observed in Lika and Gorski Kotar (0.8%) (Figure 1C).

### 3.2. Rabbit Ownership and Animal Characteristics

**Figure 2:**
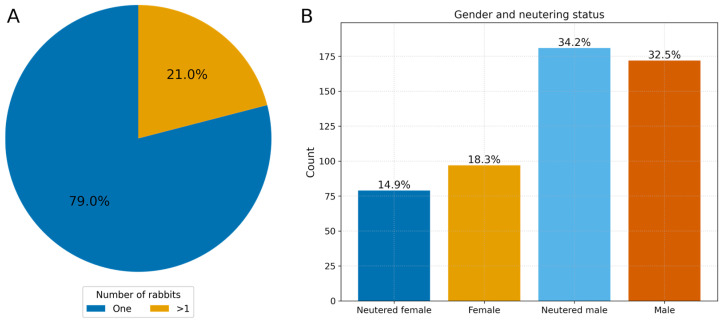
Rabbit-related characteristics, including (**A**) number of rabbits per household and (**B**) sex and neutering status. Values are presented as percentages.

Most respondents owned a single rabbit (79.0%), while 21.0% reported keeping more than one rabbit (Figure 2A).

Male rabbits were more represented overall, with similar proportions of neutered (34.2%) and intact males (32.5%). Female rabbits accounted for 33.2% of the sample, including 18.3% intact and 14.9% neutered individuals (Figure 2B).

### 3.3. Housing Conditions and Feeding Practices

Housing conditions varied, with approximately half of the rabbits kept free within the household (50.3%), while 25.1% were kept under combined housing systems. Smaller proportions were housed in pens (7.8%) or playpens (7.4%), and 9.5% were kept outdoors. Nearly all rabbits had access to free movement (96.6%), and most owners reported providing environmental enrichment (92.2%), including toys, tunnels, branches for gnawing, and other enrichment items. Mixed bedding was most commonly used (59.0%), followed by sawdust (14.5%), absorbent pads (12.5%), and hay (9.8%), whereas other materials were rarely reported. Enclosures were most frequently cleaned several times per week (46.7%), followed by daily cleaning (34.9%) and weekly cleaning (18.4%).

Feeding practices were predominantly based on mixed diets (94.9%), while only a small proportion of rabbits were fed hay alone (3.4%) or pellets alone (0.8%). Hay was reported as available to 78.2% of rabbits, whereas fresh water was available to all rabbits (Table 1).

### 3.4. Sources of Information and Owner-Reported Knowledge

**Figure 3:**
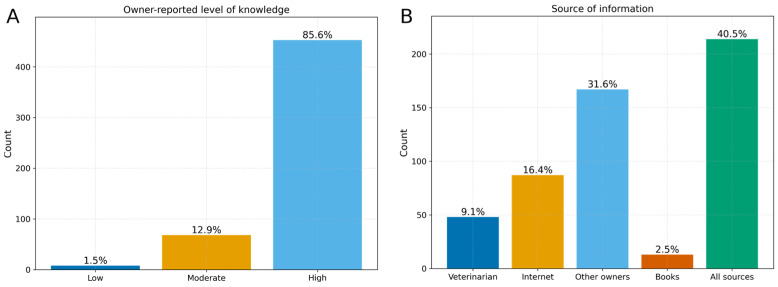
Owner-reported level of knowledge (**A**) and source of the information (**B**). Values are presented as percentages.

Most owners rated their knowledge of rabbit care as high (85.6%), whereas fewer reported moderate (12.9%) or low (1.5%) levels (Figure 3A).

Information on rabbit care was most commonly obtained from multiple sources (40.5%), followed by other rabbit owners (31.6%) and the Internet (16.4%). Veterinarians were reported as the primary source of information by only 9.1% of respondents, while books were rarely cited (Figure 3B).

### 3.5. Associations Between Owner-Reported Knowledge and Rabbit Characteristics, Housing, and Feeding Practices

**Figure 4:**
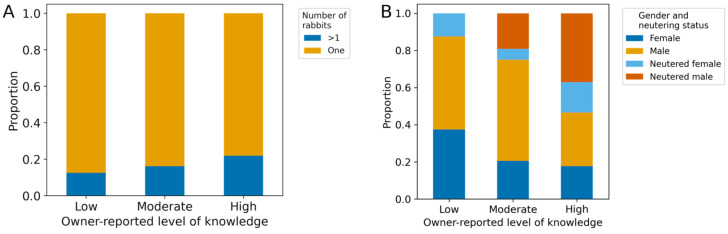
Association between owner-reported level of knowledge and (**A**) number of rabbits and (**B**) gender and neutering status of the rabbit. Data are presented as proportions of respondents within each knowledge category.

No association was observed between owner-reported knowledge and the number of rabbits owned (*p* = 0.472) (Figure 4A; Table 2). In contrast, knowledge level was significantly associated with rabbit sex and neutering status (*p* < 0.001), although the effect size was small (Cramér’s V = 0.142) (Figure 4B; Table 2).

**Figure 5:**
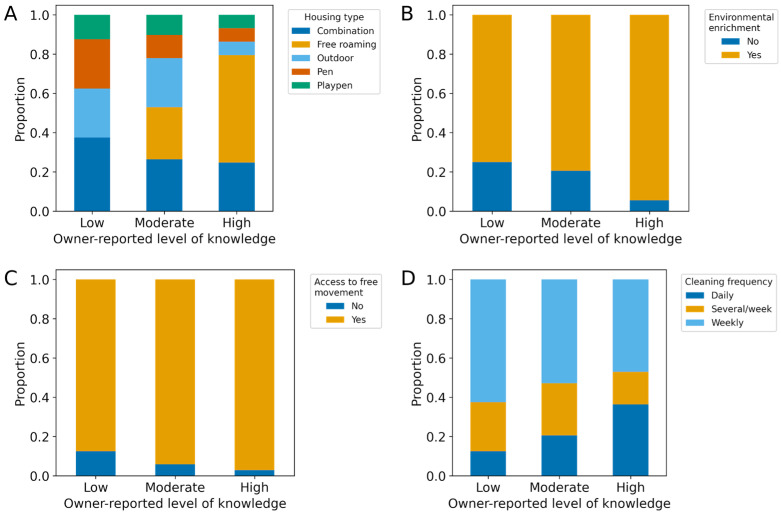
Association between owner-reported level of knowledge and (**A**) housing type, (**B**) provision of environmental enrichment, (**C**) access to free movement, and (**D**) cleaning frequency. Data are presented as proportions of respondents within each knowledge category.

Higher levels of perceived knowledge were also significantly associated with several husbandry practices, including housing type (*p* < 0.001; Cramér’s V = 0.182), environmental enrichment (*p* < 0.001; Cramér’s V = 0.195), and cleaning frequency (*p* < 0.05; Cramér’s V = 0.074) (Figure 5A,B,D, Table 2). In contrast, no significant association was found between knowledge level and access to free movement (*p* = 0.159; Cramér’s V = 0.056) (Figure 5C; Table 2).

**Figure 6:**
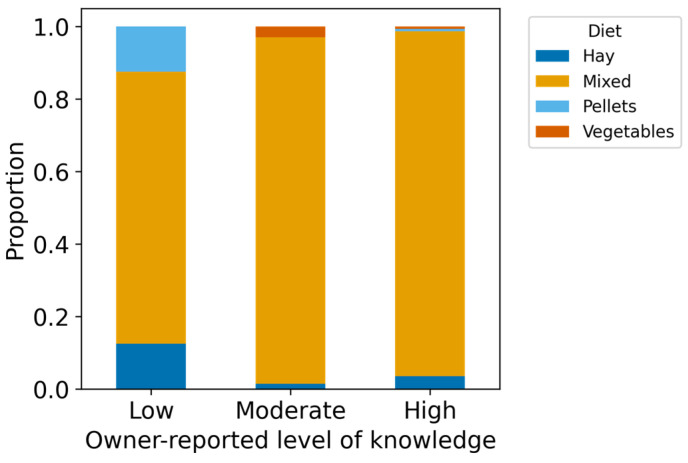
Association between owner-reported level of knowledge and diet. Data are presented as proportions of respondents within each knowledge category.

A significant association was observed between knowledge level and diet (*p* < 0.05), although the effect size was small (Cramér’s V = 0.121) (Figure 6; Table 2).

### 3.6. Association Between Owner-Reported Knowledge and Information Sources

**Figure 7:**
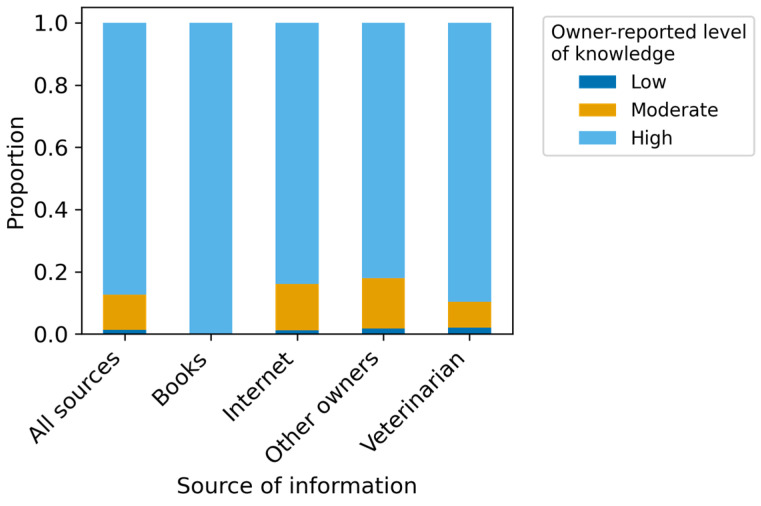
Association between the owner’s source of knowledge and the reported level of knowledge. Data are presented as proportions of respondents within each knowledge category.

No significant association was found between owner-reported level of knowledge and the primary source of information (*p* = 0.665), with a negligible effect size (Cramér’s V < 0.001) (Figure 7).

## 4. Discussion

Current rabbit welfare guidelines emphasise that appropriate husbandry should include adequate space allowance, permanent access to exercise areas, environmental enrichment, species-appropriate nutrition based predominantly on hay and/or grass, opportunities to express natural behaviours, compatible social housing, and regular veterinary care. Recommended practices further include housing that enables rabbits to run, jump, stand upright, forage, dig, and rest undisturbed, together with continuous access to fresh water and high-fibre diets [24,25,26,27,28]. Despite increasing awareness of rabbit welfare needs, previous studies continue to report substantial deficiencies in pet rabbit husbandry and welfare [3,6,29]. In line with these findings, it has been estimated that approximately 22% of pet rabbits are housed in conditions considered inadequate, most commonly in small traditional hutches that may severely restrict the performance of even basic species-specific behaviours [27].

The present study shows that, although most rabbit owners rated their knowledge of rabbit care as high (85.6%), this perceived knowledge was not consistently reflected in reported husbandry practices. Although several statistically significant associations between perceived knowledge and husbandry variables were identified, effect sizes were generally weak, suggesting that perceived knowledge alone may play only a limited role in explaining owner behaviour. Collectively, these findings indicate the presence of a knowledge–practice gap and suggest that improving rabbit welfare may require approaches that extend beyond information provision alone. Owner behaviour is likely influenced not only by knowledge but also by behavioural, motivational, and contextual factors that shape the interpretation and practical implementation of welfare recommendations [30,31,32]. Furthermore, economic considerations and spatial limitations may represent important barriers to the implementation of optimal husbandry and welfare practices.

### 4.1. Sociodemographic Characteristics of Owners

The respondent sample was strongly female-dominated, with women comprising nearly 90% of participants. This finding is consistent with previous studies reporting greater female involvement in companion animal care and higher participation of women in animal welfare-related surveys [33,34]. Nevertheless, the predominance of female respondents may also reflect participation bias commonly associated with voluntary online surveys.

The largest proportion of respondents belonged to the 26–35-year age group, which is similarly consistent with previous internet-based studies on companion animal ownership [35]. This distribution likely reflects both recruitment through social media platforms and demographic characteristics associated with this life stage, including greater independence and financial capacity for pet ownership.

More than half of the respondents originated from Zagreb and Central Croatia, potentially influencing the reported husbandry practices. Urban environments have previously been associated with more positive attitudes towards companion animals and a greater tendency to integrate pets into the household environment [36,37]. In addition, urban owners may have improved access to veterinary services, rabbit-specific products, and indoor housing opportunities, which could facilitate the implementation of certain husbandry practices. Consequently, caution is warranted when generalising these findings to less urbanised or rural populations of rabbit owners.

### 4.2. Social Housing

Rabbits are highly social animals and are generally recommended to be kept in compatible pairs or groups rather than alone [38]. Accordingly, solitary housing represents an important welfare concern, as the absence of conspecific interaction may restrict the expression of natural social behaviours and has been associated with increased fearfulness, stereotypic behaviours, and reduced lifespan [39].

A notable finding of the present study was the high proportion of rabbits kept individually (79.0%), which exceeds that reported in previous studies [6,16,35,40]. Importantly, owner-reported knowledge was not associated with the number of rabbits kept, suggesting that awareness of rabbit social needs alone may be insufficient to promote socially appropriate housing practices. This finding strongly supports the presence of a knowledge–practice gap and indicates that perceived knowledge does not necessarily translate into implementation of welfare recommendations.

It should be noted that the questionnaire specifically assessed whether rabbits were housed with other rabbits, given the importance of conspecific social interactions for rabbit welfare. Consequently, the reported proportion refers to the presence or absence of a rabbit companion and does not account for co-housing with other species, such as guinea pigs.

The persistence of solitary housing despite high self-reported knowledge may reflect the influence of practical and contextual barriers, including financial constraints, limited housing space, concerns regarding compatibility between rabbits, and reluctance to neuter animals. Consequently, decisions regarding social housing are likely shaped not only by knowledge but also by owner attitudes, perceived feasibility, and household circumstances. Improving social housing practices may therefore require behavioural and practical interventions in addition to educational approaches alone.

### 4.3. Neutering Practices

Neutering is widely recommended in pet rabbits because it facilitates social housing by reducing aggression and preventing unwanted reproduction, while also providing important health benefits, including a reduced risk of reproductive disorders [41]. In the present study, neutering was relatively common, particularly among male rabbits, which may reflect increasing awareness of its behavioural and health benefits.

Although owner-reported knowledge was significantly associated with rabbit sex and neutering status, the observed effect size was small, suggesting that perceived knowledge explained only a limited proportion of variation in neutering practices. This finding further supports the broader pattern observed throughout the study, in which knowledge alone did not consistently predict the implementation of recommended husbandry practices.

Decisions regarding neutering are likely influenced by multiple additional factors, including financial cost, access to rabbit-experienced veterinary care, owner attitudes towards surgery, and perceived anaesthetic or surgical risk. Consequently, the implementation of recommended practices may depend not only on awareness of welfare recommendations but also on the practical feasibility and acceptability of these interventions from the owner’s perspective.

### 4.4. Housing Practices

More than half of the rabbits in this study were housed in free-roaming conditions, reflecting the growing trend of keeping rabbits as integrated household companion animals. Increased space allowance and environmental complexity have been shown to promote more active and species-typical behaviours [42]. However, free-roaming housing alone should not automatically be interpreted as indicative of good welfare, as its benefits depend on appropriate environmental management, safety, and the availability of suitable resources and enrichment.

Despite increasing awareness of the benefits of free movement, previous research indicates that many pet rabbits continue to spend substantial periods confined to hutches or restricted enclosures with limited opportunities for exercise and behavioural expression [15]. Environmental enrichment aims to meet the psychological and physiological needs of animals in human care by providing essential stimuli [43]. Although more than 90% of respondents reported providing enrichment, self-reported provision does not necessarily reflect its quality, frequency, diversity, or functional value.

Higher perceived knowledge was associated with housing type and environmental enrichment, but not with actual access to free movement. This pattern suggests that perceived knowledge may be more strongly associated with visible or easily recognised aspects of care, whereas more demanding or welfare-critical practices are not consistently implemented. Collectively, these findings further support the presence of a knowledge–practice gap and indicate that owner awareness alone may be insufficient to ensure optimal housing conditions for pet rabbits.

### 4.5. Feeding Practices

Rabbits are physiologically adapted to a fibre-rich diet that is essential for maintaining gastrointestinal and dental health [6]. The predominance of mixed diets in the present study may suggest partial alignment with current feeding recommendations, which generally emphasise continuous access to hay or grass supplemented with fresh greens and limited quantities of pellets. Similar feeding patterns have been reported in broader pet rabbit populations, although previous studies also indicate that nutritionally inappropriate practices, including the provision of muesli-style diets and insufficient access to hay or fresh grass, remain relatively common [15].

However, the dietary categories used in the present study were relatively broad and should therefore be interpreted cautiously. A reported “mixed diet” does not necessarily indicate nutritional adequacy, as substantial variation may exist in the proportion, quality, and frequency of dietary components provided. Consequently, the apparent alignment with feeding recommendations may overestimate the actual adequacy of rabbit nutrition.

Although perceived knowledge was significantly associated with diet, the observed effect size was small, suggesting that knowledge alone may play only a limited role in shaping feeding practices. Given the fundamental importance of nutrition for rabbit welfare, this finding indicates that even core husbandry practices may not consistently reflect evidence-based recommendations. Feeding decisions are likely influenced by additional behavioural and contextual factors, including owner habits, convenience, commercial feeding practices, and persistent misconceptions regarding rabbit nutrition.

### 4.6. Sources of Information and Perceived Knowledge

In the present study, rabbit care information was obtained predominantly from non-professional sources despite the high levels of self-reported knowledge observed among respondents. Furthermore, no association was identified between perceived knowledge level and the primary source of information. Collectively, these findings suggest that perceived knowledge may reflect subjective confidence rather than the accuracy or depth of understanding, which may partially explain the discrepancy observed between reported knowledge and implemented husbandry practices.

These findings are consistent with previous research indicating that pet owners commonly rely on a range of information sources, including non-professional ones, and may not always accurately assess their reliability [44]. Similar patterns have also been reported among rabbit owners, who may express confidence in their understanding of rabbit care despite demonstrating limited or inaccurate knowledge regarding species-specific needs [6,17].

Importantly, misinformation or oversimplified husbandry advice may have direct welfare consequences, particularly in companion animal species whose behavioural and physiological requirements are less widely understood [19], such as rabbits.

### 4.7. Implications: Interpreting the Knowledge–Practice Gap

The present findings highlight a clear discrepancy between perceived knowledge and its practical application, indicating that deficiencies in both knowledge and husbandry practices remain widespread. Higher self-reported knowledge was primarily associated with more visible or easily implemented aspects of care, while showing little or no relationship with more complex or welfare-critical domains such as diet, social housing, and access to free movement.

This pattern suggests that perceived knowledge may reflect confidence rather than depth of understanding and that knowledge alone may be insufficient to ensure appropriate care. Instead, the implementation of recommended husbandry practices appears to be influenced by a range of behavioural and contextual factors. Consequently, current education-focused strategies that prioritise information provision may be insufficient if they do not also address behavioural and contextual barriers to implementation.

The observed discrepancy between perceived knowledge and implemented husbandry practices may cautiously be interpreted as resembling patterns previously described in relation to the Dunning–Kruger effect, a cognitive bias in which individuals with limited knowledge tend to overestimate their competence [45]. However, because the present study did not include an objective assessment of rabbit care knowledge, no direct conclusions regarding this phenomenon can be drawn.

Collectively, these findings highlight the need to move beyond knowledge dissemination towards behaviour-focused interventions, which may be more effective in improving welfare outcomes across different companion animal species.

### 4.8. Limitations of the Study and Future Directions

Several limitations should be considered when interpreting these findings. First, the study relied on self-reported data, which may be affected by recall bias, reporting bias, and social desirability bias. Second, the cross-sectional design does not allow causal relationships between perceived knowledge and husbandry practices to be established. Third, recruitment via social media represents a convenience sampling approach that may have favoured more engaged rabbit owners and contributed to the overrepresentation of female, younger, and urban respondents, thereby limiting the generalisability of the findings to the wider population. In addition, the predominance of respondents reporting high levels of knowledge may indicate a ceiling effect and may have reduced the ability to detect stronger associations between perceived knowledge and husbandry practices. The absence of an objective assessment of rabbit care knowledge further limits conclusions regarding the relationship between actual knowledge and husbandry practices. Furthermore, several husbandry variables were assessed using relatively broad categories, which may not fully reflect the quality, frequency, or adequacy of the reported practices. Finally, no direct assessments of rabbit health or welfare were performed, and the reported husbandry practices could not be objectively verified.

Future research should complement self-reported data with objective assessments of husbandry practices and direct measures of animal welfare. Longitudinal or mixed-method approaches may further improve understanding of how perceived knowledge develops over time and how it translates into practice. In addition, further studies should explore the behavioural and contextual factors influencing the implementation of husbandry practices to better inform targeted and effective educational interventions.

## 5. Conclusions

The present study identified a clear discrepancy between owner-reported knowledge and the husbandry practices of pet rabbit owners in Croatia. Even high levels of perceived knowledge did not consistently predict the implementation of appropriate husbandry practices, highlighting a potential knowledge–practice gap with direct implications for rabbit welfare, particularly in relation to social housing and feeding.

Collectively, the findings challenge the assumption that increasing owner awareness alone is sufficient to improve companion rabbit welfare. Instead, the results suggest that the practical implementation of recommended husbandry practices is likely influenced by a broader combination of behavioural, motivational, and contextual factors. Consequently, future welfare-oriented strategies may benefit from moving beyond information-based education alone towards approaches that more effectively support behavioural change, practical implementation, and informed decision-making among rabbit owners.

## Figures and Tables

**Table 1:** Owner-reported (*n* = 529) housing, feeding practices, and knowledge of pet rabbit care in Croatia.

Domain	Characteristic	Category	*n* (%)
Housing	Housing type	Free roaming	266 (50.3)
		Pen	41 (7.8)
		Playpen	39 (7.4)
		Combination	133 (25.1)
		Outdoor housing	50 (9.5)
	Access to free movement	Yes	511 (96.6)
		No	18 (3.4)
	Bedding material	Absorbent pads	66 (12.5)
		Sawdust	78 (14.5)
		Straw	14 (2.6)
		Hay	52 (9.8)
		Corn-based bedding	7 (1.3)
		Mixed bedding	312 (59.0)
	Environmental enrichment	Yes	488 (92.2)
		No	41 (7.8)
	Cleaning frequency	Daily	180 (34.9)
		Several times per week	241 (46.7)
		Weekly	95 (18.4)
Feeding	Diet type	Hay only	18 (3.4)
		Fresh vegetables only	5 (0.9)
		Pellets only	4 (0.8)
		Mixed diet	502 (94.9)
	Hay availability	Yes	413 (78.2)
		No	116 (21.8)
	Fresh water availability	Yes	529 (100.0)
		No	0 (0.0)

**Table 2:** Association between owner-reported level of knowledge and rabbit characteristics, housing, and diet.

Variable	Χ^2^	*p*-Value	Cramér’s V	Effect Size Interpretation
Number of rabbits	1.5	0.472	0.000	negligible
Gender and neutering status of the rabbit	27.2	<0.001	0.142	weak
Environmental enrichment	22.16	<0.001	0.195	weak
Housing type	42.96	<0.001	0.182	weak
Cleaning frequency	9.71	<0.05	0.074	negligible
Access to free movement	3.68	0.159	0.056	negligible
Diet	21.54	<0.05	0.121	weak

Associations were assessed using chi-squared tests. Effect sizes are reported as Cramér’s V.

## Data Availability

The original contributions presented in the study are included in the article; further inquiries can be directed to the corresponding authors.
